# Kynurenic acid ameliorates NLRP3 inflammasome activation by blocking calcium mobilization *via* GPR35

**DOI:** 10.3389/fimmu.2022.1019365

**Published:** 2022-10-13

**Authors:** Tianyin Sun, Ruiqian Xie, Hongbin He, Qianqian Xie, Xueqin Zhao, Guijie Kang, Chen Cheng, Wenwei Yin, Jingjing Cong, Jing Li, Xuefu Wang

**Affiliations:** ^1^ School of Pharmacy, Inflammation and Immune-Mediated Diseases Laboratory of Anhui Province, Anhui Medical University, Hefei, China; ^2^ School of Basic Medical Sciences, Division of Life Sciences and Medicine, University of Science and Technology of China, Hefei, China; ^3^ Institute for Viral Hepatitis, Department of Infectious Diseases, The Second Affiliated Hospital, Chongqing Medical University, Chongqing, China; ^4^ School of Life Sciences, Anhui Medical University, Hefei, China

**Keywords:** kynurenic acid, NLRP3 inflammasome, GPR35, systemic inflammation, metabolic disorder

## Abstract

The inflammasome has been linked to diverse inflammatory and metabolic diseases, and tight control of inflammasome activation is necessary to avoid excessive inflammation. Kynurenic acid (KA) is a tryptophan metabolite in the kynurenine pathway. However, the roles and mechanisms of the regulation of inflammasome activation by KA have not yet been fully elucidated. Here, we found that KA suppressed caspase-1 activation and IL-1β production in macrophages by specifically inhibiting canonical and noncanonical activation of the NLRP3 inflammasome. Mechanistically, KA reduced calcium mobilization through G-protein receptor 35 (GPR35), resulting in reduced mitochondrial damage and decreased mtROS production, thus blocking NLRP3 inflammasome assembly and activation. Importantly, KA prevented lipopolysaccharide-induced systemic inflammation, monosodium urate-induced peritoneal inflammation, and high-fat diet-induced metabolic disorder. Thus, KA ameliorated inflammation and metabolic disorders by blocking calcium mobilization-mediated NLRP3 inflammasome activation *via* GPR35. Our data reveal a novel mechanism for KA in the modulation of inflammasome activation and suggest that GPR35 might be a promising target for improving NLRP3 inflammasome-associated diseases by regulating calcium mobilization.

## Introduction

The NLRP3 inflammasome is a well-characterized member of the cytosolic inflammasome family and is composed of NEK7, NLRP3, ASC, and caspase-1 (1). Activation of the NLRP3 inflammasome requires two signals. The first signal, known as priming, stimulates Toll-like receptors (TLRs) and leads to an increase in NLRP3 and pro-IL-1β. The second signal triggers NLRP3 inflammasome assembly, leads to the cleavage of caspase-1, and finally results in the maturation and secretion of the proinflammatory cytokines IL-1β and IL-18. The activation of the NLRP3 inflammasome can be triggered by diverse stimuli, including pathogen-associated molecular patterns (PAMPs) and damage-associated molecular patterns (DAMPs) generated from pathogens, environmental stresses, metabolic disorders, and tissue damage (2). The pan-specificity of NLRP3 recognition has been explained by the interaction of NLRP3 with PtdIns4P on the dispersed trans-Golgi network (3). The NLRP3 inflammasome not only defends against pathogen infections but also contributes to multiple inflammatory tissue damage, e.g., in sepsis, type 2 diabetes (T2D), Alzheimer’s disease, and gouty arthritis (4, 5). Therefore, the activation of the NLRP3 inflammasome needs to be tightly controlled to avoid excessive detrimental inflammation.

NLRP3 inflammasome activation has been extensively investigated. Ion fluxes, including potassium (K^+^) efflux, calcium (Ca^2+^) mobilization, and chloride (Cl^–^) efflux, have been identified to be critical for NLRP3 inflammasome activation (6). K^+^ efflux is a common trigger for NLRP3 inflammasome activation, which is also required for anthrax lethal toxin-induced NLRP1b inflammasome activation (6). K^+^ efflux promotes intracellular Cl^–^ efflux and leads to mitochondrial damage and mitochondrial reactive oxygen species (mtROS) production (7). Lee et al. found that Ca^2+^ can promote the interaction of NLRP3 and ASC, although the underlying mechanisms remain unclear (8). Ca^2+^ mobilization leads to mitochondrial damage and subsequent mtDNA release and mtROS production (9). Most NLRP3 inflammasome activators induce mitochondrial damage and ROS generation. ROS facilitate the binding of thioredoxin-interacting protein (TXNIP) with NLRP3 and lead to NLRP3 inflammasome activation and IL-1β secretion (10). Alternatively, ROS oxidize mitochondrial DNA to activate the NLRP3 inflammasome (11). ROS inhibitors effectively prevent NLRP3 inflammasome activation. Altogether, although NLRP3 inflammasome activation is tightly regulated, the regulatory mechanisms underlying NLRP3 inflammasome activation remain to be completely elucidated.

Kynurenic acid (KA) is a tryptophan metabolite in the kynurenine pathway that can be produced in various types of cells and the intestinal microflora (12). The plasma levels of KA are elevated under various inflammatory conditions. The injection of LPS increases plasma levels of KA in pigs (13). Chronic social defeat increases the plasma levels of KA in mice (14). Kynurenic acid levels are also elevated in the peripheral blood of patients suffering from inflammatory bowel disease, type 2 diabetes, and multiple sclerosis (15–17). KA has been found to have immunomodulatory functions and to abrogate inflammation triggered by various stimuli. KA suppresses LPS-induced inflammation and reduces LPS-induced death in mice (18). KA is identified as an agonist of G-protein receptor 35 (GPR35), which is expressed in various populations of immune cells, such as monocytes, eosinophils, and invariant natural killer-like T (iNKT) cells (19, 20). KA stimulates lipid metabolism, increases energy utilization, and enhances anti-inflammatory gene expression in adipose tissue by activating GPR35 (21). However, the role of KA in modulating the NLRP3 inflammasome remains to be completely elucidated.

In the current study, we aimed to identify the role and mechanism of KA in modulating the NLRP3 inflammasome and NLRP3-associated diseases. We demonstrate that KA specifically prevents NLRP3 inflammasome activation by mitigating calcium mobilization and mitochondrial damage and mitochondrial ROS production *via* GPR35, which improves inflammatory diseases and metabolic disorders. Our findings provide new insights into the role of the tryptophan metabolite KA in controlling inflammation and suggest that GPR35 is a potential target of KA for the treatment of NLRP3-associated inflammatory diseases by regulating calcium modulation.

## Materials and methods

### Mice and ethics

C57BL/6J mice (sex: male; weight: 20-22 g; age: 6-8 weeks) were purchased from Shanghai Laboratory Animal Center, Chinese Academy of Sciences. *Nlrp3^–/–^* mice were kindly provided by Professor Rongbin Zhou (University of Science and Technology of China, Hefei, China). *Gpr35^–/–^* mice (C57BL/6J background) were purchased from Bioraylab Medicine Company (Shanghai, China). All mice were housed in groups of no more than five mice per cage and maintained in a specific pathogen-free, temperature- and light-controlled environment (22.5°C and 42.5% humidity, under a 12-hour/12-hour light–dark cycle) with free access to food and water at the animal facilities of Anhui Medical University. Mice were maintained on an irradiated sterile diet and provided autoclaved water. All experiments were performed according to the Guide for the Care and Use of Laboratory Animals. The study was approved by the Local Ethics Committee for Animal Care and Use at Anhui Medical University.

### Reagents

Kynurenic acid (K3375, purity≥98%) was obtained from Sigma−Aldrich company (Missouri, USA). M-CSF was obtained from NOVUS (Colorado, USA). LPS, nigericin, ATP, MSU, Pam3CSK4, PMA, and *poly(A/T)* were obtained from Sigma−Aldrich (Missouri, USA). MitoTracker Red, MitoSOX and Lipofectamine 2000 were obtained from Invitrogen (California, USA). Flou-4 AM and DAPI were obtained from Beyotime (Shanghai, China). The *Salmonella typhimurium* strain ATCC-14028 was obtained from Beina Chuanglian Biotech Co., Ltd. (Beijing, China).

### Cell preparation and stimulation

Bone marrow-derived macrophages were collected and cultured as previously described (22). Briefly, bone marrow cells were isolated from tibia and femoral bone marrow cells and cultured for 4 to 6 days in DMEM supplemented with 10% FBS, 1% penicillin−streptomycin solution, and 20 ng·ml^–1^ M-CSF. To induce NLRP3 inflammasome activation, 5 × 10^5^·ml^–1^ BMDMs were plated overnight in 12-well plates, and the medium was replaced with opti-MEM the following morning. BMDMs were primed with 50 ng·ml^–1^ LPS or 400 ng·ml^–1^ Pam3CSK4 (for noncanonical inflammasome activation) for 2 hours and then were treated with KA for another 2 hours. After that, the cells were stimulated with MSU (150 μg·ml^–1^) or *Salmonella typhimurium* (multiplicity of infection) for 4 hours with ATP (2.5 mM) or nigericin (4 μM) for 30 minutes. Cells were also transfected with *poly(A/T)* (0.5 μg·ml^–1^) for 4 hours or with LPS (500 ng·ml^–1^) for 16 hours by using Lipofectamine 2000. The precipitated supernatants and cell extracts were collected for immunoblotting and ELISA analyses. LDH release was detected using an LDH Cytotoxicity Assay Kit (Beyotime, Shanghai, China). THP-1 cells (ATCC Cat# TIB-202, RRID: CVCL_0006) were treated with 50 ng·ml^–1^ PMA for 3 hours, differentiated into macrophages, and then used to investigate the activation of the NLRP3 inflammasome in human macrophages.

### Immunoblotting

The sample buffer was added to the cell lysates and boiled at 100°C for 10 minutes. The same amount of protein was loaded into SDS−PAGE gel wells. The gel was electrophoresed at 80 V for 0.5 hours and 120 V for 1 hour. Proteins were transferred from gels onto PVDF membranes (Millipore, Massachusetts, USA) at 90 V for 1 hour. The membranes were blocked with 5% nonfat milk at room temperature for 2 hours and then incubated overnight with the following primary antibodies in primary antibody dilution buffer at 4°C: anti-IL-1β (R&D Systems Cat# AF-401-NA, RRID: AB_416684), anti-caspase-1 (AdipoGen Cat# AG-20B-0042, RRID: AB_2490248), anti-NLRP3 (AdipoGen Cat# AG-20B-0014, RRID: AB_2490202), anti-ASC (Cell Signaling Technology Cat# 67824, RRID: AB_2799736), anti-human IL-1β (Cell Signaling Technology Cat# 83186, RRID: AB_2800010), anti-human caspase-1 (Cell Signaling Technology Cat# 3866, RRID: AB_2069051), and anti-β-actin (ZSGB-Bio Cat# TA-09, RRID: AB_2636897). The membrane was washed three times with PBST for 5 minutes each and then incubated for 1 hour at room temperature using conjugated secondary antibodies (1:5000) in blocking buffer. The membrane was washed three times with PBST for 5 minutes each time. Images were obtained after chemiluminescence visualization.

### ELISA

The detection of cytokines, including mouse IL-1β (R&D, Minnesota, USA), IL-18 (Invitrogen, California, USA), TNF-α (R&D, Minnesota, USA) and human IL-1β (Dekewe, Shanghai, China) as well as human TNF-α (Dekewe, Shanghai, China), in cell culture supernatants or sera was performed according to the manufacturer’s instructions.

### ASC oligomerization assay

After stimulation with nigericin (4 μM), BMDMs were washed with ice-cold PBS, and NP-40 lysis buffer (Beyotime, Shanghai, China) was then added to lyse cells for 30 minutes at 4°C. The samples were centrifuged at 350 × g for 10 minutes at 4°C. The pellets were rinsed with ice-cold PBS three times and resuspended in 500 μl of PBS. The pellet suspensions were crosslinked for 30 minutes with 2 mM disuccinimidyl suberate (Sangon Biotech, Shanghai, China) at room temperature. Next, the samples were centrifuged for 10 minutes at 350 × g at 4°C. The crosslinked pellets were mixed with 30 μl of sample buffer and then subjected to immunoblot analysis.

### Intracellular potassium determination

To measure the intracellular potassium (K^+^) concentration, the supernatants of BMDMs stimulated with NLRP3 inflammasome-activating stimuli in 6-well plates were removed, and the cells were washed with K^+^-free buffer (139 mM NaCl, 1.7 mM NaH_2_PO_4_, and 10 mM Na_2_HPO_4_, pH 7.2). The cells were lysed with HNO_3_ and boiled at 100°C for 30 minutes. Then, distilled water was added to dissolve the precipitated products. The intracellular K^+^ concentration was measured by inductively coupled plasma−optical emission spectrometry with a PerkinElmer Optima 7300 DV spectrometer (PerkinElmer, USA).

### Cytosolic Ca^2+^ detection

BMDMs were seeded at a density of 1×10^5^ cells per well in 96-well plates and cultured overnight. After LPS priming, cells were loaded with 1 µM Fluo 4-AM (Beyotime, Shanghai, China) and 0.05% (w/v) Pluronic F-127 for 30 minutes; each well was briefly washed three times with HBSS (Ca^2+^-free) and replaced with HBSS (Ca^2+^-containing). The plate was placed into a Cytation 5 cell imaging multimode reader (BioTek, USA) preheated to 37°C. Baseline fluorescence (488 nm excitation and 520 nm emission at 20-second intervals) was recorded for 6 minutes. Then, the cells were stimulated with nigericin. The fluorescence of the cells was quantified with measurements at 20-second intervals. Fluorescence intensities were normalized to obtain the fold increase in intensity.

### Immunoprecipitation

For endogenous immunoprecipitation detection, BMDMs plated in 6-well plates were stimulated to activate the NLRP3 inflammasome and lysed with NP-40 lysis buffer comprising complete protease inhibitor. The cell lysates were rotated and incubated overnight with the primary antibodies and protein A/G-agarose (Abmart, Shanghai, China) at 4°C. The antibody-bound proteins were precipitated with protein A/G beads, and immunoblot analysis was performed.

### Immunofluorescence

For immunofluorescence, 2-3×10^5^·ml^–1^ BMDMs were plated on coverslips overnight and then stimulated and stained with MitoTracker Red (50 nM) or MitoSox (5 μM). After washing with PBS three times, the cells were fixed with 4% paraformaldehyde at room temperature for 20 minutes and then washed with PBST three times. DAPI was used for nuclear counterstaining. Confocal microscopy analyses were carried out using a ZEISS LSM 800 (ZEISS, Germany).

### LPS-induced sepsis

To induce sepsis, mice were injected intraperitoneally (i.p.) with KA (50 mg·kg^–1^) or an equal volume of PBS at 24 hours and 2 hours before intraperitoneal injection of LPS (20 mg·kg^–1^). After 4 hours, the serum was collected, the mice were euthanized, and IL-1β, IL-18 and TNF-α were detected by ELISA.

### MSU-induced peritonitis

To induce peritonitis, mice were injected intraperitoneally (i.p.) with KA (50 mg·kg^–1^) or PBS at 24 hours and 2 hours before intraperitoneal injection of MSU (1 mg of MSU crystals dissolved in 0.5 ml of sterile PBS). After 6 hours, the mice were euthanized, and their peritoneal cavities were subjected to lavage with 10 ml of ice-cold PBS. Peritoneal exudate cells (PECs) were isolated, counted, and assessed by flow cytometry for analysis of neutrophil recruitment. IL-1β production in peritoneal lavage fluid was determined by ELISA.

### High-fat diet and KA treatment

To induce metabolic inflammation, mice were fed a normal diet (ND) or a high-fat diet (HFD) for 14 weeks. Mice at 8 weeks of HFD feeding were treated with KA at a dose of 50 mg·kg^–1^ by oral administration every other day or an equal volume of saline for 6 weeks. During KA treatment and the subsequent experiments, HFD feeding of mice was maintained.

### Glucose tolerance test and insulin tolerance test

Glucose tolerance tests (GTTs) were performed *via* intraperitoneal injection of glucose at 1.5 g·kg^–1^ after 14 hours of fasting. Insulin tolerance tests (ITTs) were performed *via* intraperitoneal injection of human recombinant insulin (Novo Nordisk) at a dose of 0.75 U·kg^–1^ after 4 hours of fasting. Blood glucose levels were measured from the tail veil at 0, 15, 30, 60, and 90 minutes after glucose or insulin injection.

### Tissue culture

The isolated adipose and liver tissues were washed twice in PBS, minced into fine pieces, and cultured in 12-well plates (0.5 g·well^–1^) in M199 medium supplemented with 5% FBS and 1% penicillin and streptomycin. After 24 hours, the culture supernatants were analysed by ELISA for IL-1β, IL-18, and TNF-α.

### Statistical analyses

The values are expressed as the mean ± s.e.m. The unpaired t test or nonparametric Mann−Whitney test (GraphPad Software) was used for statistical analysis. Data were considered significant when p ≤ 0.05 (*), p≤ 0.01 (**), or p ≤ 0.005 (***).

## Results

### KA inhibits NLRP3 inflammasome activation

**Figure 1:**
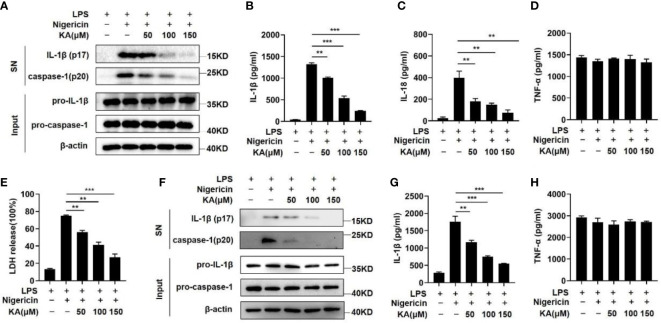
KA suppressed NLRP3 inflammasome-mediated caspase-1 activation and IL-1β secretion. LPS-primed BMDMs or THP-1 cells were pretreated with KA at different concentrations for 2 hours and then stimulated with nigericin. **(A)** Western blot analysis of IL-1β and cleaved caspase-1 (p20) in the supernatants (SN) of BMDMs and western blot analysis of pro-IL-1β and pro-caspase-1 in the lysates (input) of BMDMs. **(B)** ELISA of IL-1β in the SN of BMDMs. **(C)** ELISA of IL-18 in the SN of BMDMs. **(D)** ELISA of TNF-α in the SN of BMDMs. **(E)** Lactate dehydrogenase (LDH) levels in the SN of BMDMs after nigericin-induced pyroptosis. **(F)** Western blot analysis of IL-1β and cleaved caspase-1 (p20) in the SN of THP-1 cells and of pro-IL-1β and pro-caspase-1 in the lysates (input) of THP-1 cells. **(G)** ELISA of IL-1β in the SN of THP-1 cells. **(H)** ELISA of TNF-α in the SN of THP-1 cells. Data are representative of three independent experiments and show as the mean ± SEM. Statistical significance was analysed by unpaired t test: **p < 0.01, ***p < 0.001.

To examine the effects of KA on NLRP3 inflammasome activation, lipopolysaccharide (LPS)-primed mouse bone marrow-derived macrophages (BMDMs) were pretreated with the indicated dose of KA before stimulation with the NLRP3 agonist nigericin. We found that KA suppressed nigericin-induced caspase-1 cleavage and IL-1β maturation in a dose-dependent manner (**Figure 1A**). Furthermore, KA suppressed the secretion of IL-1β and IL-18 (**Figures 1B, C**). However, the expression of the inflammasome-independent cytokine TNF-α was not impaired by KA (**Figure 1D**). Given that NLRP3 inflammasome activation causes macrophage pyroptosis, we detected LDH release in the supernatant of BMDMs at 2 hours after nigericin treatment. We found that KA mitigated nigericin-induced BMDM pyroptosis (**Figure 1E**), further suggesting the inhibitory effect of KA on NLRP3 inflammasome activation. To test the role of KA in NLRP3 activation in THP-1 cells, THP-1 cells were treated with KA before nigericin stimulation. Caspase-1 activation and IL-1β, but not TNF-α, secretion in THP-1 cells were suppressed by KA in a dose-dependent manner (**Figures 1F–H**), suggesting that KA also inhibits the activation of the NLRP3 inflammasome in THP-1 cells. Collectively, these results suggest that KA inhibits the activation of the NLRP3 inflammasome.

### KA specifically inhibits NLRP3 inflammasome activation

**Figure 2:**
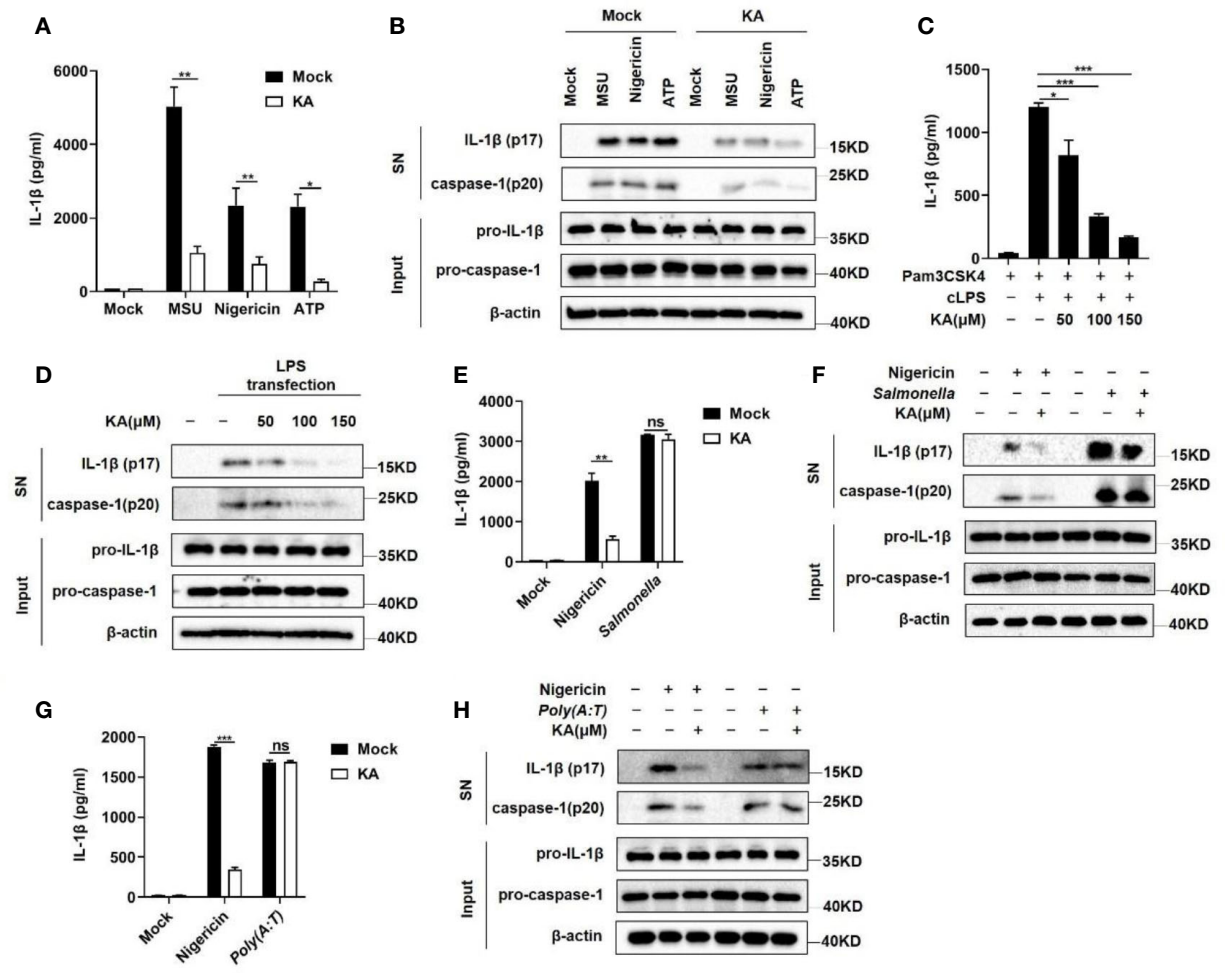
KA specifically suppressed the NLRP3 inflammasome. **(A, B)** LPS-primed BMDMs were pretreated with or without KA (150 μM) for 2 hours and then stimulated with MSU, nigericin, or ATP. Mock denotes stimulation with PBS. ELISA of IL-1β in the SN of BMDMs **(A)** and western blot analysis of IL-1β and cleaved caspase-1 in the SN of BMDMs and pro-IL-1β and pro-caspase-1 in the lysates (input) of BMDMs **(B)**. **(C, D)** Pam3CSK4-primed BMDMs were treated with KA for 2 h and then stimulated with cytoplasmic LPS by transfection for 16 h. ELISA of IL-1β in the SN of BMDMs **(C)** and western blot analysis of IL-1β and cleaved caspase-1 in the SN of BMDMs and pro-IL-1β and pro-caspase-1 in the lysates (input) of BMDMs **(D)**. **(E, F)** LPS-primed BMDMs were treated with KA for 2 hours and then stimulated with nigericin for 30 minutes or subjected to *Salmonella typhimurium* infection for 4 hours. ELISA of IL-1β in the SN of BMDMs. **(E)** Western blot analysis of IL-1β and cleaved caspase-1 in the SN of BMDMs and pro-IL-1β and pro-caspase-1 in the lysates (input) of BMDMs **(F)**. **(G, H)** LPS-primed BMDMs were treated with KA for 2 hours and then stimulated with nigericin for 30 minutes or subjected to *poly(dA:dT)* transfection for 4 hours. ELISA of IL-1β in the SN of BMDMs **(G)** and western blot analysis of IL-1β and cleaved caspase-1 in the SN of BMDMs and pro-IL-1β and pro-caspase-1 in the lysates (input) of BMDMs **(H)**. Data are representative of three independent experiments and show as the mean ± SEM. Statistical significance was analysed by unpaired t test: *p < 0.05, **p < 0.01, ***p < 0.001; ns, no significance.

Given that the NLRP3 inflammasome can be activated by a variety of stimuli, we examined the effect of KA on the activation of the NLRP3 inflammasome triggered by other stimuli. The results showed that KA suppressed caspase-1 cleavage and IL-1β secretion induced by MSU and ATP, similar to nigericin (**Figures 2A, B**). Moreover, KA blocked caspase-1 cleavage and IL-1β secretion induced by the activation of the nonclassical inflammasome triggered by cytoplasmic LPS (**Figures 2C, D**). To determine the specificity of the inhibitory effect of KA on the NLRP3 inflammasome, we examined the role of KA in the activation of the NLRC4 and AIM2 inflammasomes. The results showed that KA had no effect on *Salmonella typhimurium* infection-induced NLRC4 or *poly (dA:dT)* transfection-induced AIM2 inflammasome activation (**Figures 2E–H**). Collectively, these results suggest that KA specifically inhibits the activation of the NLRP3 inflammasome.

### KA inhibits NLRP3 inflammasome assembly, Ca^2+^ signalling, mitochondrial damage, and mtROS production

**Figure 3:**
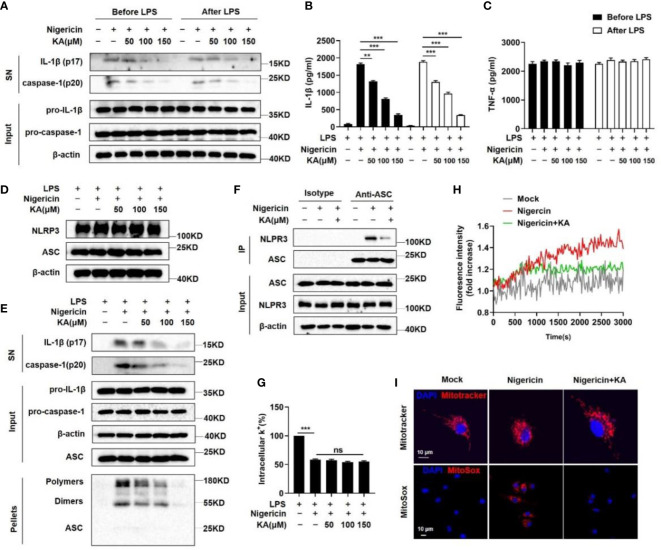
KA inhibits NLRP3 inflammasome assembly, Ca^2+^ signalling, mitochondrial damage and mtROS production. **(A-C)** BMDMs were treated with KA and then stimulated with LPS (KA before LPS), or BMDMs were treated with LPS and then stimulated with KA (KA after LPS). After that, the cells were stimulated with nigericin. Western blot analysis of IL-1β and cleaved caspase-1 in the SN of BMDMs and pro-IL-1β and pro-caspase-1 in the lysates (input) of BMDMs **(A)**, ELISA of IL-1β **(B)** and TNF-α in the SN of BMDMs **(C)**. **(D)** LPS-primed BMDMs were pretreated with KA for 2 hours and then stimulated with nigericin. Western blot analysis of NLRP3 and ASC in the lysates (input) of BMDMs. **(E)** Western blot analysis of IL-1β and cleaved caspase-1 in the SN of BMDMs, pro-IL-1β and pro-caspase-1 in the lysates (input) of BMDMs, and crosslinked ASCs in the NP-40-insoluble pellet of BMDMs pretreated with or without KA. **(F)** Immunoprecipitation and western blot to evaluate the NLRP3-ASC interaction in BMDMs pretreated with or without KA. **(G)** Quantification of potassium efflux in BMDMs pretreated with the indicated doses of KA. **(H)** Intracellular calcium in nigericin-stimulated BMDMs pretreated with or without KA was analysed by a Cytation 5 cell imaging multimode reader to measure fluorescence intensity. **(I)** LPS-primed BMDMs were treated with KA and then stimulated with nigericin, followed by staining with MitoTracker red or MitoSox. Confocal microscopy analysis of MitoTracker (red) or MitoSOX (red) in BMDMs. Bars (10 µm) are in white, and nuclei (DAPI) are in blue. Data are representative of three independent experiments and show as the mean ± SEM. Statistical significance was analysed by unpaired t test: **p < 0.01, ***p < 0.001; ns, no significance.

To explore the mechanism by which KA inhibits NLRP3 inflammasome activation, we first examined the role of KA in NLRP3 inflammasome priming. BMDMs were treated with KA before LPS stimulation. We found that treatment with KA before LPS stimulation was able to suppress caspase-1 cleavage and IL-1β maturation but failed to suppress the levels of LPS-induced pro-IL-1β and TNF-α (**Figures 3A–C**). Furthermore, it has been reported that the activation of the NLRP3 inflammasome can be inhibited by decreasing the protein levels of NLRP3 or ASC *via* autophagy as well as ubiquitination and proteasomal degradation (23, 24); however, our results showed that KA did not affect the protein levels of NLRP3 and ASC (**Figure 3D**). Together, these results suggest that KA suppresses the activation of the NLRP3 inflammasome without affecting LPS-induced NF-κB-dependent priming of the NLRP3 inflammasome. Thus, we then detected the formation of ASC oligomerization, which is a critical step in NLRP3 inflammasome assembly and activation (25). KA blocked nigericin-induced ASC oligomerization in a dose-dependent manner (**Figure 3E**). The recruitment of ASC to NLRP3 is another critical step for NLRP3 inflammasome complex formation (26). The nigericin-induced endogenous NLRP3–ASC interaction was substantially suppressed by KA (**Figure 3F**), indicating that KA acts upstream of ASC oligomerization to inhibit NLRP3 inflammasome assembly and activation.

Potassium efflux and calcium mobilization act as early upstream signals of NLRP3 inflammasome assembly and trigger NLRP3 inflammasome activation (27). Therefore, we identified the effect of KA on these upstream signals of NLRP3 inflammasome activation. The results showed that KA failed to prevent the decrease in intracellular potassium in BMDMs stimulated with nigericin (**Figure 3G**), suggesting that KA does not affect potassium efflux. In contrast, the nigericin-induced increase in intracellular calcium levels was inhibited by KA (**Figure 3H**), suggesting that KA prevents Ca^2+^ mobilization to suppress NLRP3 inflammasome activation. Moreover, it has been reported that Ca^2+^ mobilization triggers mitochondrial damage, leading to the production of mitochondrial ROS (mtROS) and NLRP3 inflammasome activation (9). Therefore, we next tested whether KA affected mitochondrial damage and mtROS production during NLRP3 inflammasome activation and found that KA efficiently suppressed mitochondrial damage and mtROS production (**Figure 3I**). To confirm these findings, we next detected mitochondrial damage and mtROS production by flow cytometry. Consistently, the results showed that KA decreased the frequency of BMDMs with mitochondrial damage and reduced mtROS production in BMDMs (**Supplementary Figures 1A, B**). Taken together, these findings show that KA blocks NLRP3 inflammasome assembly and activation by suppressing Ca^2+^ mobilization and subsequent mitochondrial damage as well as mtROS production in BMDMs.

### KA inhibits NLRP3 inflammasome activation *via* GPR35

**Figure 4:**
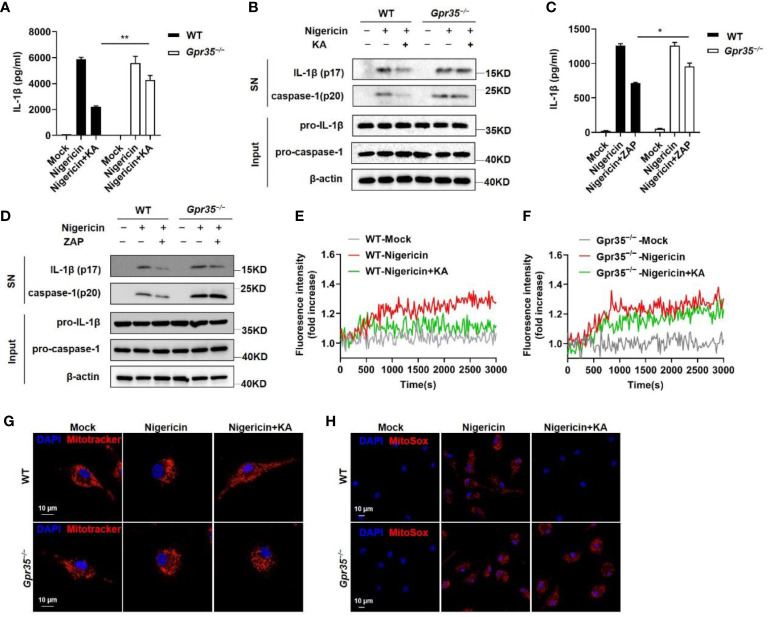
KA inhibited NLRP3 inflammasome activation *via* GPR35. **(A, B)** LPS-primed BMDMs from WT and *Gpr35^−/−^* mice were treated with KA and then stimulated with nigericin. ELISA of IL-1β in the SN of BMDMs (a) and western blot analysis of IL-1β and cleaved caspase-1 in the SN of BMDMs and pro-IL-1β and pro-caspase-1 in the lysates (input) of BMDMs (b). **(C, D)** LPS-primed BMDMs from WT and *Gpr35^−/−^* mice were treated with ZAP and then stimulated with nigericin. ELISA of IL-1β in the SN of BMDMs **(C)** and western blot analysis of IL-1β and cleaved caspase-1 in the SN of BMDMs and pro-IL-1β and pro-caspase-1 in the lysates (input) of BMDMs **(D)**. **(E, F)** LPS-primed BMDMs from WT and *Gpr35^−/−^* mice were treated with or without KA and then stimulated with nigericin. Intracellular calcium in WT and Gpr35^−/−^ BMDMs was analysed with a Cytation 5 cell imaging multimode reader cell to measure fluorescence intensity. **(G, H)** LPS-primed WT and *Gpr35^−/−^* BMDMs were treated with or without KA (150 µM) and then stimulated with nigericin, followed by staining with MitoTracker red or MitoSox. Confocal microscopy analysis of MitoTracker in red **(G)** or MitoSox in red **(H)** in WT and *Gpr35^−/−^* BMDMs. Bars (10 µm) are in white, and nuclei (DAPI) are in blue. **(I)** LPS-primed WT and *Gpr35^−/−^* BMDMs were treated with KA and then stimulated with nigericin for 30 minutes. Cells were stained with MitoTracker green and MitoTracker red, and the frequency of MitoTracker green^+^red^+^ cells was detected by flow cytometry and statistically analysed. **(K)** LPS-primed WT and *Gpr35^−/−^* BMDMs were treated with KA and then stimulated with nigericin for 30 minutes. Cells stained with MitoSox were analysed by flow cytometry, and the mean fluorescence intensity (MFI) was statistically analysed. Data are representative of three independent experiments and show as the mean ± SEM. Statistical significance was analysed by unpaired t test: *p < 0.05, **p < 0.01.

The finding that KA failed to directly bind with Ca^2+^ suggests that KA could not suppress Ca^2+^ mobilization by itself (**Supplementary Figure 2A**). KA has been reported to be an agonist of GPR35 (19). Therefore, we investigated whether KA inhibited the activation of the NLRP3 inflammasome *via* GPR35. The results showed that the absence of GPR35 impaired the KA-induced inhibition of IL-1β secretion and caspase-1 cleavage (**Figures 4A, B**). Consistently, the human GPR35 antagonist ML145 abrogated KA-mediated suppression of NLRP3 inflammasome activation in THP-1 cells (**Supplementary Figure 2B**), suggesting that GPR35 mediated the KA-induced inhibition of NLRP3 inflammasome activation. To confirm the role of GPR35 in the inhibition of the NLRP3 inflammasome, a synthetic agonist of GPR35, zaprinast (ZAP), was used. The results showed that ZAP inhibited IL-1β secretion and caspase-1 cleavage. ZAP-mediated inhibition was partially abrogated in the absence of GPR35 (**Figures 4C, D**). We further determined whether GPR35 was required for the KA-mediated inhibition of Ca^2+^ signalling and subsequent mitochondrial injury and mtROS production. The results showed that the absence of GPR35 impaired the KA-mediated inhibition of Ca^2+^ signalling and subsequent mitochondrial injury and mtROS production (**Figures 4E–H**). To confirm these findings, we detected mitochondrial damage and mtROS production by flow cytometry. Consistently, the absence of GPR35 impaired the KA-mediated inhibition of mitochondrial damage and mtROS production in BMDMs (**Supplementary Figures 1C, D**). Collectively, these results indicate that KA inhibits NLRP3 inflammasome activation *via* GPR35.

### KA mitigates NLRP3 inflammasome-mediated inflammatory diseases

**Figure 5:**
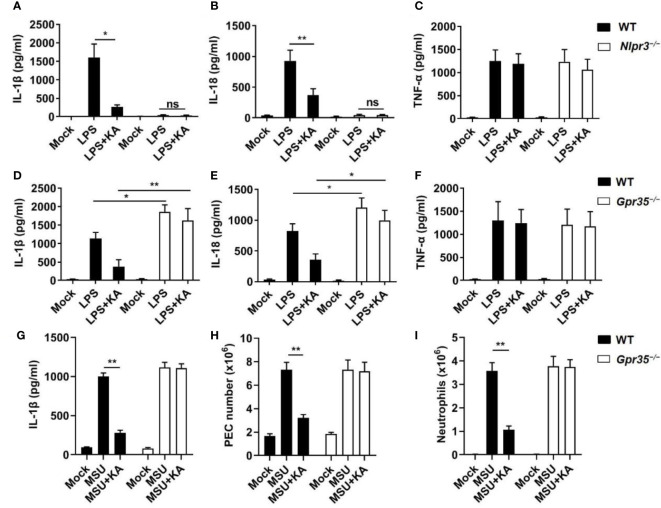
KA mitigates NLRP3 inflammasome-mediated inflammatory diseases. **(A-C)** ELISA of IL-1β **(A)**, IL-18 **(B)**, and TNF-α **(C)** in serum from WT and *Nlrp3^−/−^* mice intraperitoneally injected with LPS (20 mg·kg^−1^ body weight) with or without KA (50 mg·kg^−1^ body weight). **(D-F)** ELISA of IL-1β **(D)**, IL-18 **(E)**, and TNF-α **(F)** in serum from WT and *Gpr35^−/−^* mice intraperitoneally injected with LPS (20 mg·kg*^−^* ^1^ body weight) with or without KA (50 mg·kg^−1^ body weight). **(G-I)** ELISA of IL-1β **(G)**, the peritoneal exudate cell (PEC) number **(H)**, and neutrophil number **(I)** in the peritoneal cavity from WT and *Gpr35^−/−^* mice intraperitoneally injected with MSU crystals (1 mg·mouse^−1^) in the presence or absence of KA (50 mg·kg^−1^). Data are representative of three independent experiments and show as the mean ± SEM. Statistical significance was analysed by the nonparametric Mann−Whitney test: *p < 0.05, **p < 0.01; ns, no significance.

To explore the therapeutic potential of KA *in vivo*, we examined whether KA ameliorated NLRP3-mediated inflammatory diseases. We first investigated the effect of KA on LPS-induced sepsis, which is a typical model of NLRP3-driven inflammation (28). As expected, the results showed that serum IL-1β and IL-18 levels were significantly decreased in wild-type mice treated with KA but not in *Nlrp3^−/−^* mice (**Figures 5A, B**). In contrast, KA had little effect on serum TNF-α production (**Figure 5C**). These results suggest that KA can inhibit LPS-induced NLRP3 inflammasome activation and sepsis. To identify whether KA improved LPS-induced sepsis *via* GPR35, WT and *Gpr35^−/−^* mice were challenged with LPS in the presence or absence of KA. The results showed that the serum levels of IL-1β and IL-18 were significantly increased in *Gpr35^−/−^* mice compared with those in WT mice (**Figures 5D, E**). KA inhibited LPS-induced serum IL-1β and IL-18 in WT mice but failed to suppress the LPS-induced increases in IL-1β and IL-18 in *Gpr35^−/−^* mice (**Figures 5D, E**). KA had no effect on serum TNF-α levels (**Figure 5F**). KA improved LPS-induced tissue damage (**Supplementary Figures 3A, B**). Moreover, KA treatment significantly increased the survival of mice challenged with LPS (**Supplementary Figure 3C**). To further confirm the inhibitory effect of KA on inflammation, peritoneal injection of MSU was used to induce NLRP3-dependent peritonitis (29). KA significantly alleviated MSU-induced IL-1β production and peritoneal exudate cell (PEC) and neutrophil recruitment in WT mice but not *Gpr35^−/−^* mice (**Figures 5G–I**). KA did not influence MSU-induced IL-6 or TNF-α production (**Supplementary Figures 4A, B**), suggesting that KA ameliorates MSU-induced NLRP3-mediated peritoneal inflammation *in vivo*. Collectively, these results indicate that KA inhibits inflammatory diseases *via* suppression of NLRP3 inflammasome activation through GPR35.

### KA mitigates NLRP3 inflammasome-mediated metabolic disorder

**Figure 6:**
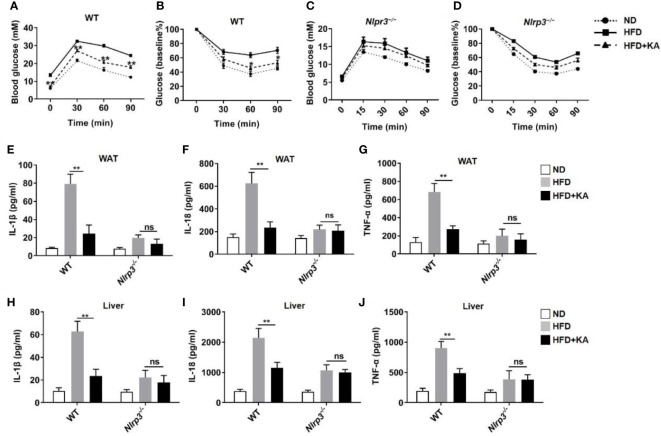
KA mitigates NLRP3 inflammasome-mediated metabolic disorder. Mice were fed a normal diet (ND) or a high-fat diet (HFD) for 14 weeks. Mice at 8 weeks of HFD feeding were treated with KA at a dose of 50 mg·kg^–1^ by oral administration every other day or an equal volume of saline for 6 weeks. **(A, B)** Glucose tolerance **(A)** and insulin tolerance **(B)** were tested in WT mice. **(C, D)** Glucose tolerance **(C)** and insulin tolerance **(D)** were tested in *Nlrp3^−/−^* mice. **(E-G)** Adipose tissue (WAT) was isolated and cultured for 24 hours, and supernatants were analysed by ELISA for the release of IL-1β **(E)**, IL-18 **(F)**, and TNF-α **(G)**. **(H, I)** The livers were isolated and cultured for 24 hours, and the supernatants were analysed by ELISA for the release of IL-1β **(H)**, IL-18 **(I)**, and TNF-α **(J)**. Data are representative of three independent experiments and show as the mean ± SEM. Statistical significance was analysed by the nonparametric Mann−Whitney test: p < 0.05, **p < 0.01; ns, no significance.

The NLRP3 inflammasome contributes to the progression of metabolic disorders (30). To determine whether KA ameliorates metabolic disorders by inhibiting the activation of the NLRP3 inflammasome, we fed mice a high-fat diet (HFD) with or without KA supplementation for 14 weeks and then performed a glucose tolerance test (GTT) and insulin tolerance test (ITT). Wild-type mice fed a HFD developed glucose intolerance and insulin resistance, whereas KA treatment markedly improved glucose tolerance and insulin sensitivity (**Figures 6A, B**). *Nlrp3^−/−^* mice were resistant to HFD-induced metabolic disorders. The therapeutic effects of KA on metabolic disorders were not observed in HFD-fed *Nlrp3^−/−^* mice (**Figures 6C, D**). To further confirm the effect of KA on metabolic stress-induced inflammasome activation, adipose and liver tissues were isolated for culture. Consistent with the above results, the production of IL-1β and IL-18 in adipose and liver tissue from HFD-treated mice was higher than that in mice on a normal diet (**Figures 6E, F, H, I**). KA administration reduced HFD-induced IL-1β or IL-18 production in these tissues (**Figures 6E, F**, **H, I**), indicating that KA suppresses metabolic stress-induced activation of the NLRP3 inflammasome. In addition, KA inhibited the upregulation of TNF-α in wild-type mice (**Figures 6G, J**), indicating that KA plays an extensive anti-inflammatory role. Taken together, our results demonstrate that KA protects against metabolic disorders *via* inhibition of the NLRP3 inflammasome.

## Discussion

**Figure 7:**
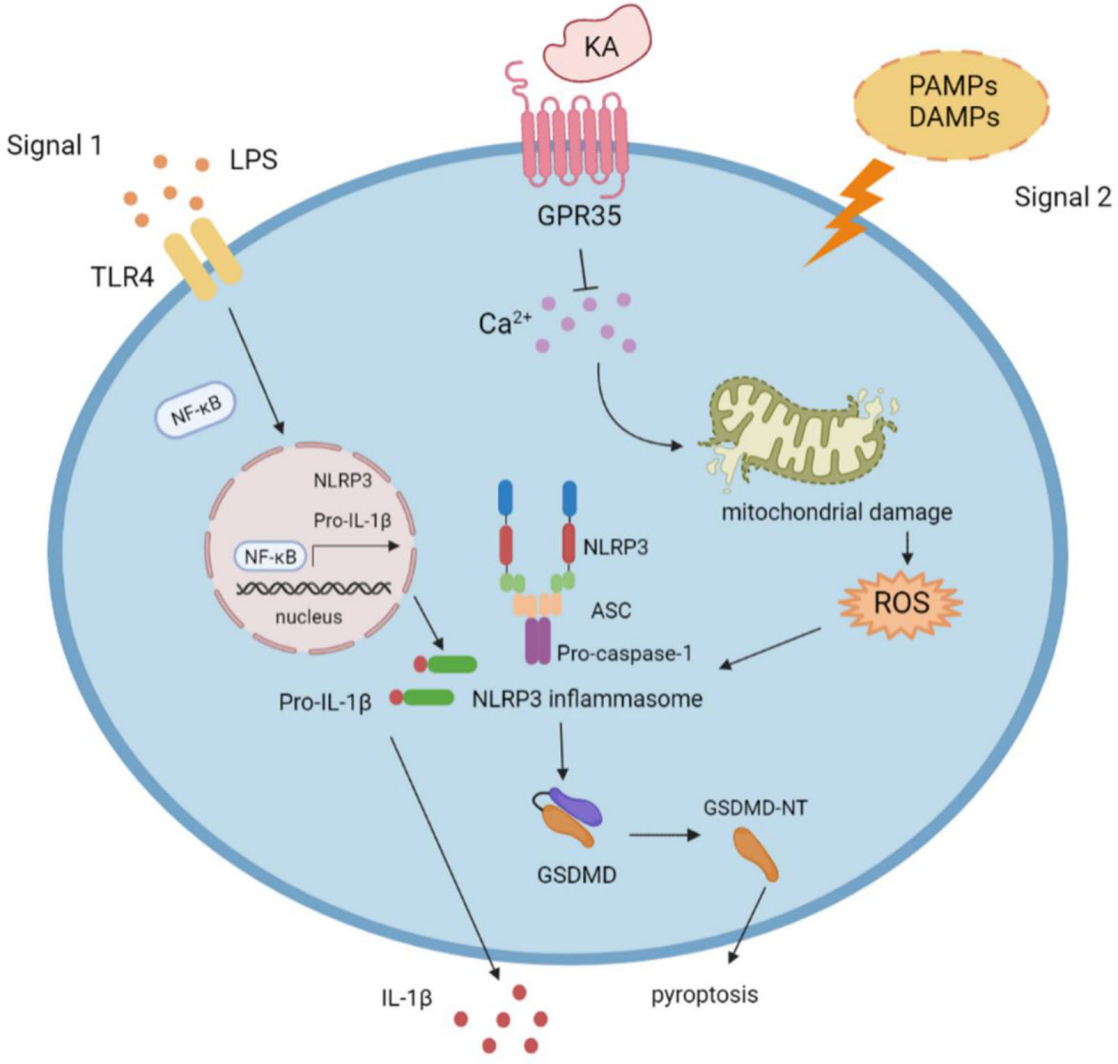
Model for KA-induced suppression of NLRP3 activation. Following the NLRP3 inflammasome priming step (signal 1), NLRP3-activating stimuli (signal 2) trigger NLRP3 inflammasome assembly and activation. KA suppresses NLRP3 inflammasome activation and NLRP3-associated diseases by suppressing calcium mobilization, reducing mitochondrial damage and mitochondrial ROS production, and blocking NLRP3 inflammasome assembly *via* GPR35.

The NLRP3 inflammasome responds to numerous stimuli *via* secretion of IL-1β and IL-18 and by inducing pyroptotic death responses. However, overactivation of the NLRP3 inflammasome leads to various inflammatory diseases and tissue damage. Therefore, tight regulation of NLRP3 inflammasome activation is needed to avoid a detrimental inflammatory response. In this study, we report that the tryptophan metabolite KA specifically inhibits NLRP3 inflammasome activation, prevents IL-1β-mediated inflammation and gasdermin D-mediated pyroptosis by targeting GPR35 and then ameliorates NLRP3-associated inflammatory diseases (**Figure 7**), suggesting that KA is a candidate for suppressing NLRP3-mediated inflammation and that GPR35 is a potential target for NLRP3-related disorders.

Numerous synthetic compounds have been identified as potent inhibitors of the NLRP3 inflammasome and have been tested in animal models. However, only a few endogenous compounds in hosts have been reported to inhibit the NLRP3 inflammasome. KA is an endogenous tryptophan metabolite in the kynurenine pathway that possesses immunomodulatory activity. KA induces IL-6 in the presence of inflammatory signalling *via* the aryl hydrocarbon receptor (AHR) (31). KA reduces the expression of IL-23 in LPS-stimulated dendritic cells and subsequently inhibits Th17 cell differentiation *in vitro* (32). In addition, KA reduces high-mobility group box 1 (HMGB1) protein secretion in monocytes and reduces IL-4 secretion in iNKT cells (33, 34). Our data demonstrated that KA blocked caspase-1 cleavage and IL-1β secretion induced by both canonical and noncanonical NLRP3 inflammasome activators in a dose-dependent manner, indicating that KA is a broad-spectrum inhibitor of the NLRP3 inflammasome. KA had no influence on NLRC4 and AIM2 inflammasome-mediated caspase-1 cleavage and IL-1β secretion, suggesting that KA specifically suppresses the NLRP3 inflammasome. Further analysis showed that the KA-mediated inhibition of the NLRP3 inflammasome occurred at the activation but not the priming step, given that KA blocked the assembly of the NLRP3 inflammasome but had no effect on LPS-induced NLRP3 and pro-IL-1β, which is further supported by the finding that pretreatment with KA before LPS suppressed IL-1β but not TNF-α production. Consistent with this result, the findings of Hao et al. showed that KA deactivates the NLRP3 inflammasome and reduces IL-1β secretion (35). They reported that KA promotes autophagy-mediated NLRP3 degradation. In our study, however, we found that KA had no influence on NLRP3 protein levels. The discrepancy in the mechanisms by which KA inhibits the NLRP3 inflammasome needs further analysis. GPR35 is a known endogenous receptor of KA (19). Considering the species orthologue selectivity, GPR35 antagonists were not used to identify KA-dependent receptors in our study. We confirmed that the KA-induced inhibition of the NLRP3 inflammasome partially depends on GPR35 in macrophages from GPR35-knockout mice. KA activates AHR and hydroxycarboxylic acid receptor 3 (HCAR3) (36, 37). AHRs are potential receptors of KA, which have also been reported to inhibit the NLRP3 inflammasome *via* suppressing the transcription of NLRP3 and reducing protein levels of NLRP3 (38). The protein levels of NLRP3 were comparable in KA-treated macrophages and controls in our study. We speculated that the AHR-mediated reduction in NLRP3 levels might not contribute to the KA-induced inhibition of NLRP3 inflammasome activation. However, the roles of AHR and other potential receptors of KA in the inhibition of the NLRP3 inflammasome need further investigation. Moreover, KA at high concentrations acts as a potent endogenous antioxidant (39), which might play a role in suppressing NLRP3 inflammasome activation. In addition, ZAP still has an effect on IL-1β production in GPR35-knockout BMDMs. ZAP can inhibit phosphodiesterases (PDEs) (40). PDE4 inhibition suppresses NLRP3 inflammasome activation (41). It was speculated that ZAP might suppress NLRP3 inflammasome activation by inhibiting PDEs other than activating GPR35. Although this information might be helpful to explain the partial inhibition of NLRP3 inflammasome activation by KA and ZAP in the absence of GPR35, further investigation is required to identify the reasons.

NLRP3 inflammasome assembly is essential for its activation. We found that KA inhibits nigericin-induced ASC oligomerization and the NLRP3-ASC interaction, leading to the inhibition of NLRP3 inflammasome activation. Ion fluxes and mitochondria play critical roles in triggering NLRP3 inflammasome assembly. Accumulating evidence suggests that the disturbance of intracellular ions orchestrates NLRP3 inflammasome activation. Potassium efflux has been recognized to be necessary and sufficient for NLRP3 inflammasome assembly. Increased intracellular Ca^2+^ levels due to calcium mobilization may lead to Ca^2+^ overload in the mitochondria, resulting in impaired mitochondrial function, the production of mtROS, and the release of mtDNA (42). Therefore, crosstalk between ion fluxes and mitochondria occurs during NLRP3 inflammasome activation. Our results revealed that KA inhibits calcium mobilization and prevents nigericin-induced mitochondrial damage and mitochondrial ROS production. In contrast, KA failed to modify nigericin-induced potassium and chloride efflux. It has been reported that GPR35 activation can reduce Ca^2+^ mobilization (43), which also contributes to the KA-mediated amelioration of mitochondrial damage and mtROS production in BMDMs. In addition to GPR35-mediated signals, KA itself acts as an antioxidant and ROS scavenger (39), which might directly reduce mitochondrial ROS and inhibit NLRP3 inflammasome activation. Therefore, the mechanisms by which KA prevents mitochondrial damage and mtROS production need further investigation.

KA supplementation improved LPS-induced sepsis, MUS-induced peritoneal inflammation, and HFD-induced insulin resistance *in vivo*. Moreover, the therapeutic function of KA *in vivo* depends on its inhibitory effect on the NLRP3 inflammasome. Consistent with our findings, KA has been found to ameliorate HFD-induced weight gain and glucose tolerance by increasing energy utilization and anti-inflammatory gene expression (21). Moreover, KA decreased the levels of IL-1β, IL-18 and TNF-α. NLRP3 inhibition has no direct influence on TNF-α production in *in vitro* cell models and *in vivo* acute inflammatory diseases. However, in chronic inflammation, IL-1β and IL-18 can induce the production of other proinflammatory cytokines, such as TNF-α and MCP-1 (44). Thus, NLRP3 inhibition by KA reduced the production of IL-1β and IL-18 and then led to a reduction in TNF-α. It has also been reported that NLRP3 inflammasome inhibition prevents Western diet-induced cardiac hypertrophy, fibrosis and atherosclerosis (45–47). Whether KA administration improves Western diet-induced cardiac hypertrophy, fibrosis and atherosclerosis remains unknown. Furthermore, it remains unclear whether the KA effect in this model is mediated by suppression of NLRP3 inflammasome activation. Given that overactivation of the NLRP3 inflammasome leads to inflammatory diseases such as Alzheimer’s disease, gout, and atherosclerosis (48), KA or other agonists of GPR35 might have potential as new therapeutics for NLRP3-triggered diseases. Moreover, it has been reported that KA has neuroprotective, anticonvulsant, antiatherogenic, antiulcerative, analgesic, and hepatoprotective effects in addition to anti-inflammatory properties (48, 49). However, the potential side effects of KA on the host must be identified and avoided when using KA in the treatment of NLRP3-triggered diseases. Taken together, we found that KA specifically inhibits NLRP3 inflammasome activation through GPR35 and represents a potential therapeutic agent for NLRP3-related inflammatory diseases.

## Data availability statement

The original contributions presented in the study are included in the article/**Supplementary Material**. Further inquiries can be directed to the corresponding authors.

## Ethics statement

The animal study was reviewed and approved by the Local Ethics Committee for Animal Care and Use at Anhui Medical University. Written informed consent was obtained from the owners for the participation of their animals in this study.

## Author contributions

TS, RX, and HH conducted experiments and analyzed data. QX, XZ, GK and CC assisted the experiments and data analysis. WY and JC revised the paper. JL and XW conceptualized and supervised the study, designed experiments, and wrote the manuscript. All authors contributed to the article and approved the submitted version.

## Funding

This work was supported by the National Natural Science Foundation of China (grant numbers 31872741) and by Anhui Provincial Natural Science Foundation (grant numbers 2108085Y28) and by Research Improvement Program of Anhui Medical University (grant numbers 0601066205).

## Conflict of interest

The authors declare that the research was conducted in the absence of any commercial or financial relationships that could be construed as a potential conflict of interest.

## Publisher’s note

All claims expressed in this article are solely those of the authors and do not necessarily represent those of their affiliated organizations, or those of the publisher, the editors and the reviewers. Any product that may be evaluated in this article, or claim that may be made by its manufacturer, is not guaranteed or endorsed by the publisher.
