# Supplementary material for: Kynurenic acid ameliorates NLRP3 inflammasome activation by blocking calcium mobilization via GPR35
Source: Front Immunol. 2022 Oct 13;13:1019365. doi: 10.3389/fimmu.2022.1019365 (PMC9606686; doi:10.3389/fimmu.2022.1019365)
Supplement: Supplementary file 1 [file DataSheet_1.docx]

Supplementary Material

Materials and methods

Assessment of tissue damage

The damage to the liver, lung and kidney was evaluated by histological analyses. Tissue specimens were fixed in 4% paraformaldehyde, dehydrated, and embedded in paraffin. Liver sections were prepared and stained with haematoxylin and eosin for histochemical evaluation. Liver injury was also evaluated based on the serum levels of alanine aminotransferase (ALT) and aspartate aminotransferase (AST). ALT and AST levels were measured using a diagnostic kit (Mindray Bio, Shenzhen, China).

Induction and detection of pyroptosis

BMDMs were incubated with 1 μg/ml LPS for 4 hours and subsequently replaced with 10 μM nigericin for pyroptotic induction for 2 hours, followed by detection of the LDH release rate. Pyroptosis was assessed by the lactate dehydrogenase (LDH) assay using an LDH kit (Beyotime, Shanghai, China). To detect the effect of KA on pyroptosis, KA at the indicated dose was added to LPS-stimulated BMDMs 2 hours before nigericin treatment.

Binding assay of KA and calcium ions in a free cell system

A calcium assay kit (Abcam, Massachusetts, UK) was used to detect the binding of KA and calcium ions in a free cell system. Briefly, after the sample was prepared, the reaction mixture was added and incubated for 5 minutes. Finally, the optical density at 575 nm was measured on a microplate reader. The calcium concentration was then calculated according to the standard curve.

Mitochondrial staining

For mitochondrial staining, 3×10^5^·ml^–1^ BMDMs were plated on nontreated cell culture plates overnight and then stimulated and stained with MitoTracker green (Invitrogen, California, USA) (50 nM), MitoTracker red (50 nM) or MitoSox (5 μM). The changes in mitochondrial membrane potential and mitochondrial ROS were analysed by flow cytometry.

Survival rates

The survival rate of WT and Gpr35^−/−^ mice was observed after intraperitoneal injection of a lethal dose of LPS (30 mg·kg^–1^ body weight). Mice were pretreated with or without KA by intraperitoneal injection at 24 hours and 2 hours before LPS treatment.

Figures and legends


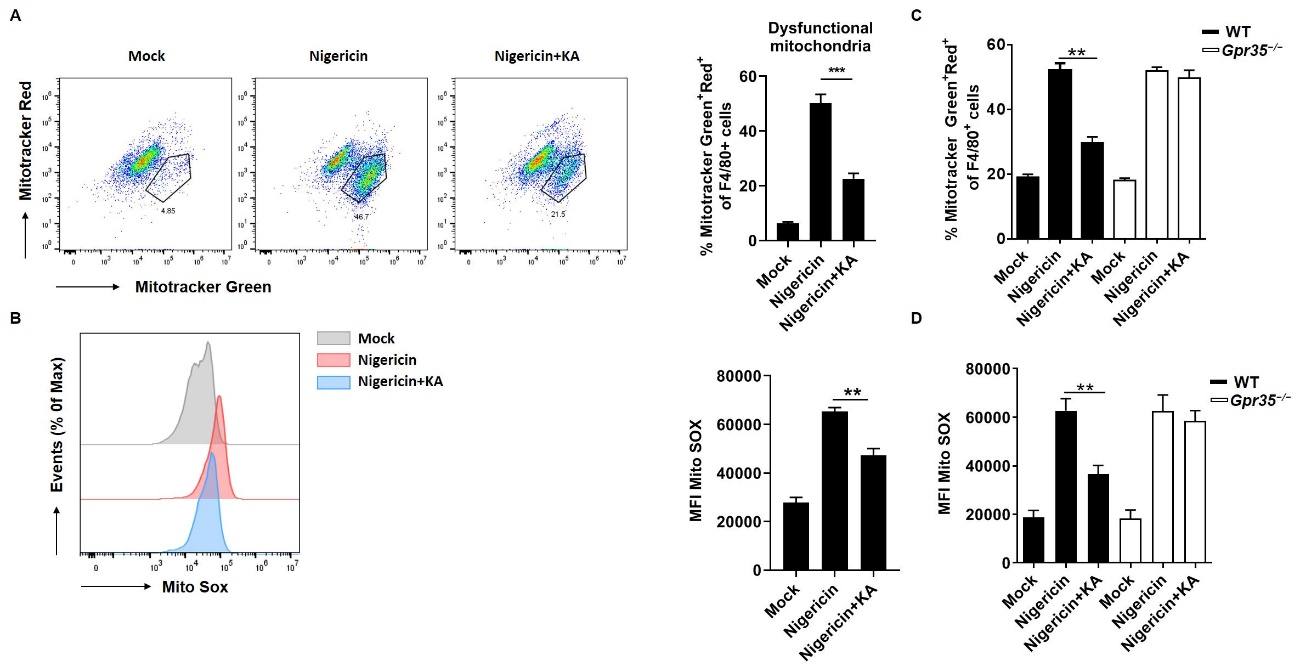


Supplementary Figure 1. KA inhibits nigericin-induced mitochondrial damage and mtROS production. (A) LPS-primed BMDMs were treated with KA and then stimulated with nigericin for 30 minutes. Cells were stained with MitoTracker green and MitoTracker red, and the frequency of MitoTracker green^+^ red^+^ cells was detected by flow cytometry and statistically analysed. (B) LPS-primed BMDMs were treated with KA and then stimulated with nigericin for 30 minutes. Cells stained with MitoSox were analysed by flow cytometry, and the mean fluorescence intensity (MFI) was statistically analysed. (C) LPS-primed BMDMs from WT and *Gpr35^–/–^* mice were treated with KA and then stimulated with nigericin for 30 minutes. Cells were stained with MitoTracker green and MitoTracker red, and the frequency of MitoTracker green^+^ red^+^ cells was detected by flow cytometry and statistically analysed. (D) LPS-primed BMDMs from WT and *Gpr35^–/–^* mice were treated with KA and then stimulated with nigericin for 30 minutes. Cells stained with MitoSox were analysed by flow cytometry, and the mean fluorescence intensity (MFI) was statistically analysed. Data are representative of three independent experiments and show as the mean ± SEM. Statistical significance was analysed by unpaired t test: *p < 0.05, **p < 0.01, ***p < 0.001.


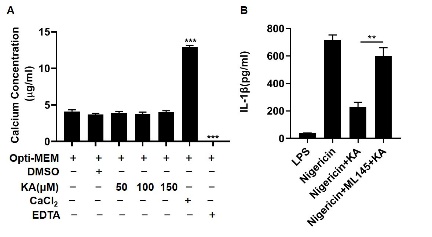


Supplementary Figure 2. KA fails to bind with calcium ions and inhibit NLRP3 inflammasome activation in the presence of ML145. (A) The combination of KA and calcium was analysed with a calcium assay kit, and the fluorescence intensity was measured. (B) LPS-primed PMA-activated THP-1 cells were treated with ML145 (10 μM) for 30 minutes before pretreatment with KA (150 μM) for 2 hours and then were stimulated with nigericin. IL-1β in the SN of THP-1 cells was detected by ELISA. Data are representative of three independent experiments and show as the mean ± SEM. Statistical significance was analysed by unpaired t test: *p < 0.05, **p < 0.01, ***p < 0.001.


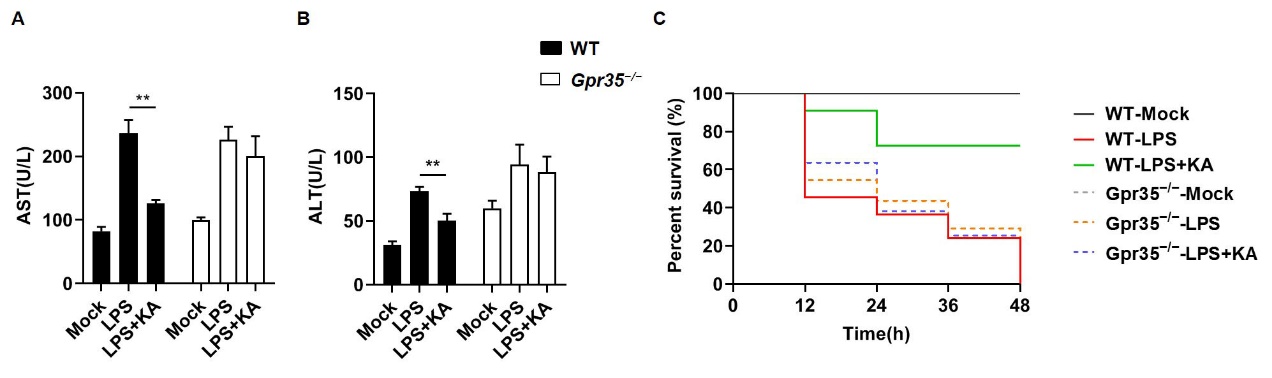


Supplementary Figure 3. KA inhibits tissue damage and improves mortality in sepsis. (A-B) ALT (A) and AST (B) in serum from WT and Gpr35^−/−^ mice intraperitoneally injected with LPS (20 mg·kg^−1^ body weight) with or without KA (50 mg·kg^−1^ body weight). (C) The survival rate of WT and Gpr35^−/−^ mice pretreated with or without KA after injection of a lethal dose of LPS (30 mg·kg^−1^ body weight). Data are representative of three independent experiments and show as the mean ± SEM. Statistical significance was analysed by the nonparametric Mann‒Whitney test: p < 0.05, **p < 0.01, ***p < 0.001.


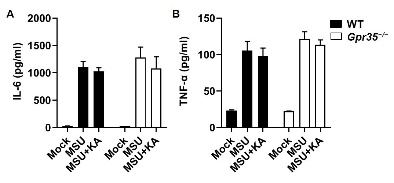


Figure S4. KA had no effect on IL-6 and TNF-α in MSU-induced peritonitis. (A-B) ELISA of IL-1β (A) and TNF-α (B) in the peritoneal cavity from WT and Gpr35^−/−^ mice intraperitoneally injected with MSU crystals (1 mg·mouse^−1^) in the presence or absence of KA (50 mg·kg^−1^). Data are representative of three independent experiments and show as the mean ± SEM. Statistical significance was analysed by the nonparametric Mann‒Whitney test: p < 0.05, **p < 0.01, ***p < 0.001.
